# High-throughput analysis using non-depletive SPME: challenges and applications to the determination of free and total concentrations in small sample volumes

**DOI:** 10.1038/s41598-018-19313-1

**Published:** 2018-01-18

**Authors:** Ezel Boyacı, Barbara Bojko, Nathaly Reyes-Garcés, Justen J. Poole, Germán Augusto Gómez-Ríos, Alexandre Teixeira, Beate Nicol, Janusz Pawliszyn

**Affiliations:** 1 Department of Chemistry, University of Waterloo, 200 University Avenue West, Waterloo, Ontario, N2L 3G1 Canada; 2 Unilever U.K., Safety and Environmental Assurance Centre, Colworth Science Park, Sharnbrook Bedford, MK441LQ United Kingdom; 3 Present Address: Department of Chemistry, Middle East Technical University, Ankara, 06800 Turkey; 4 Present Address: Department of Pharmacodynamics and Molecular Pharmacology, Faculty of Pharmacy, Collegium Medicum in Bydgoszcz, Nicolaus Copernicus University in Toruń, 85-067 Bydgoszcz, Poland

## Abstract

*In vitro* high-throughput non-depletive quantitation of chemicals in biofluids is of growing interest in many areas. Some of the challenges facing researchers include the limited volume of biofluids, rapid and high-throughput sampling requirements, and the lack of reliable methods. Coupled to the above, growing interest in the monitoring of kinetics and dynamics of miniaturized biosystems has spurred the demand for development of novel and revolutionary methodologies for analysis of biofluids. The applicability of solid-phase microextraction (SPME) is investigated as a potential technology to fulfill the aforementioned requirements. As analytes with sufficient diversity in their physicochemical features, nicotine, N,N-Diethyl-meta-toluamide, and diclofenac were selected as test compounds for the study. The objective was to develop methodologies that would allow repeated non-depletive sampling from 96-well plates, using 100 µL of sample. Initially, thin film-SPME was investigated. Results revealed substantial depletion and consequent disruption in the system. Therefore, new ultra-thin coated fibers were developed. The applicability of this device to the described sampling scenario was tested by determining the protein binding of the analytes. Results showed good agreement with rapid equilibrium dialysis. The presented method allows high-throughput analysis using small volumes, enabling fast reliable free and total concentration determinations without disruption of system equilibrium.

## Introduction

Non-depletive determinations of compounds in biological matrices play a significant role in many bioanalytical studies (e.g., pharmacokinetic, toxicokinetic, protein binding determinations, etc.)^1,2^. Barring a few exceptions, most current bioanalytical assays seek protocols that are applicable to relatively small sample volumes (e.g. 100 µL)^3^. For instance, methods that enable high-throughput analysis, while only requiring one hundred microliters of biofluid or less, would be highly desirable in a hospital environment where many samples are generated, fast determination and diagnosis are imperative, and only small samples can be taken from certain patients, such as newborns. Further, recent paradigm shifts^4–9^ in the toxicological risk assessment arena have spurred the demand for characterization of the exposure in *in vitro* assay systems to enable non animal-based risk assessments^10,11^, a challenge currently being faced by many industries. While nominal concentrations of chemicals added to test systems have long served as the basis of potency calculations due to the accessibility of the nominal concentrations, in many cases, nominal concentrations poorly reflect actual biologically effective doses. In an *in vitro* assay system, chemicals may differentially bind to culture media constituents (e.g. serum albumin), as well as undergo non-specific binding with cells and sampling apparatus, such as the walls of well-plates. They may also undergo evaporation, degradation, or metabolic changes at different rates throughout the exposure period necessary for analysis. Such processes, in turn, may reduce the bioavailable, and thus, biologically effective dose of test chemicals in *in vitro* assays to levels far below their nominal concentration. In this context, employment of methods capable of measuring free and total concentrations over time in *in vitro* assay systems would aid in the interpretation of *in vitro* test results for extrapolation to *in vivo* conditions.

However, many sampling/sample-preparation methods can disturb the existing equilibrium in the system under study; thus, in investigations concerning free and total concentrations, erroneous conclusions may be derived if the implemented sample preparation method has not been properly designed^12,13^. In response to the aforementioned limitations of bioanalytical studies, many researchers have begun to show greater interest in solid phase microextraction (SPME) based techniques, primarily due to their flexibility as an extractive tool, their compatibility with sampling modes and systems (e.g., *in vivo*, *in vitro*, small sample volumes, etc.)^14–17^, and their easily automated sample preparation procedures. In addition to the progressive adoption of the most commonly known and accepted SPME geometry (wherein the fiber is coated with a layer of extraction phase) towards a variety of applications, there has been growing interest in the development of applications that use the thin-film SPME (TF-SPME) format, which is designed to enhance the sensitivity of SPME-based assays via its moderately larger surface (for example, a blade) and relatively larger extraction volumes^18^. Further, the introduction of the TF-SPME brush coupled to an automated sample preparation station has enabled high-throughput extraction and sample preparation for this geometry of SPME, further expanding its applicability towards a variety of bioanalytical assays. To date, numerous TF-SPME studies using the Concept 96 system have been published; in this regard, in addition to reporting the attainment of high extraction recoveries, such studies have reported remarkably shorter processing times per sample as compared to traditional methods, largely owing to the ability of the station to handle 96 samples simultaneously^19,20^. The use of fibers for relatively large sample volumes (1–2 mL), as well as for smaller volumes (e.g. 100 µL), is well-documented^21–23^. Similarly, typical commercial TF-SPME blade applications have been reported to employ sample volumes in the range of 1–1.5 mL. Further decreases in final sample volume are limited by the size and geometry of the used extraction phase^19,20,24,25^. Recently, total concentrations in small sample volumes (down to a few microliters) have been successfully measured by employing miniaturized SPME fibers coated with polypyrrole, and by spotting samples on TF-SPME devices^26^. However, in systems where the ratio of extraction phase volume to sample volume is moderately high, significant deviations from non-depletive conditions may occur, potentially resulting in a disturbance of the existing analyte-matrix-binding equilibrium as it moves towards the analyte-extraction-phase equilibrium. Thus, there currently exists a demand for the development of non-depletive sampling devices applicable to small sample volumes (e.g. 100 µL) that would enable the time-course monitoring of the dynamics and kinetics of a given system, such as the monitoring of free and/or total concentrations in a test system over time.

In this work, two configurations of solid-phase microextraction, namely thin film coated blades and fibers, were evaluated with regards to their applicability to address all of the aforementioned criteria while also providing fast, high-throughput, non-depletive, and simultaneous determination of free and total concentrations of various drugs in biological matrices. To this end, TF-SPME blades and SPME fibers with short coating lengths and various chemistries were prepared and tested for their applicability in a small sample volume (one hundred microlitres) context. Likewise, coating chemistries were characterized and evaluated with regards to their suitability for determination of time-course concentrations of compounds of different physicochemical properties in typical 96-well plate *in vitro* assay test systems.

## Materials and Methods

### Chemicals and solutions

Standards used in this experiment included (±) nicotine, diclofenac, and N,N-Diethyl-meta-toluamide (DEET); (±) nicotine and diclofenac were purchased from Sigma (Oakville, ON, Canada), while N,N-Diethyl-meta-toluamide (DEET) was acquired from Fluka (Oakville, ON, Canada). Internal standards (IS) used in this study included (±) nicotine-d_4_, N,N-Diethyl-3-methyl-d_3_-benzamide-2,4,5,6-d_4_ (DEET-d_7_), and diclofenac-d_4_ (phenyl-d_4_-acetic), all of which were purchased from CDN isotopes (Pointe-Claire, QC, Canada). Mobile phases and desorption solutions were prepared from LC grade acetonitrile (ACN) and methanol (MeOH), obtained from Sigma-Aldrich. Finally, mobile phase additives, LC-MS grade ammonium acetate, ammonium fluoride, and formic acid were supplied by Fluka (Oakville, ON, Canada).

TF-SPME blades and ultra-thin coated SPME fibers were prepared in-house. Details regarding the TF-SPME coating procedure are provided in the Supplementary Information, which can be found online. Ultra-thin coated SPME fibers were also prepared using an in-house coating method. C18 particles (5 µm in particle diameter), hydrophilic-lipophilic-balanced particles (HLB; polymeric reversed-phase; 60 µm in particle diameter), and pentafluorophenylpropyl (F5) (PFP; 3 µm in diameter), used for preparation of TF-SPME coatings, were kindly provided by Supelco. Polystyrene-divinyl benzene with a weak anion exchanger (PS-DVB-WAX, Chromabond Sorbent Easy) was obtained from Macherey-Nagel.

Polyacrylonitrile (PAN) with an average molecular weight of 150 000, obtained from Aldrich (Oakville, ON, Canada), was dissolved in N,N-dimethylformamide (Sigma-Aldrich, Oakville, ON, Canada) and used as a glue to immobilize functional particles on the surface of the supports. 2 mL Nunc U96 deep well plates, 0.39 mL VWR tissue culture plates (96 wells-F; sterile), and 0.2 mL VWR PCR plates (96 wells; skirted) were purchased from VWR International (ON, Canada) and used for SPME protocols. Fetal calf serum (sterile-filtered, cell culture tested) obtained from Sigma, was used for investigations performed in the presence of a binding matrix.

With the exception of nicotine-d_4_, which was obtained as a ready-to-use 0.1 mg mL^−1^ solution in ACN, standard and internal standard stock solutions were prepared at a concentration of 1.0 mg mL^−1^ in MeOH, while lower concentrations of the mixed-analyte stock solutions were prepared weekly. Working (extraction) solutions were prepared in phosphate buffer saline (PBS, pH 7.4), in calf serum (CS), and in 5% calf serum (CS) diluted in PBS, keeping the amount of organic solvent in the final sample at less than 1% (v/v). All standard solutions were stored at −30 °C until required for use.

### Instrumentation

A Thermo TSQ Vantage (triple quadrupole) mass spectrometer equipped with a heated electrospray ionization (H-ESI) ion source was used under selected reaction monitoring (SRM) conditions. Positive ion mode was used to monitor nicotine and DEET, while diclofenac was monitored in negative ion mode. The selected spray voltage was 1.0 kV, and the vaporizer and transfer capillary temperatures were set at 275 °C. Sheath and auxiliary gas (N_2_) pressures were 40 AU and 15 AU, respectively. Argon was used as a collision gas, and kept at a pressure of 1.5 mTorr. The optimized tuning parameters for each compound are shown in Supplementary Table S1. The liquid chromatography system consisted of a Thermo Accela pump equipped with an on-line vacuum degasser and a Thermo Accela autosampler. For separation of nicotine and nicotine-d_4_, an Ascentis Express HILIC column (10 cm × 2.1 mm; 2.7 µm; Supelco) was used. Chromatographic separation was achieved using solvent A, which consisted of 10.0 mM of ammonium acetate in water, with a pH adjusted to 3.0 using formic acid; and solvent B, which was comprised of ACN with a 300 µL min^−1^ flow rate. Separation of diclofenac, DEET, diclofenac-d_4_, and DEET-d_7_ was achieved with an Ascentis Express F5 column (5 cm × 2.1 mm; 2.7 µm; Supelco), using1.0 mM ammonium fluoride in water as solvent A, and ACN as solvent B, and a flow rate of 300 µL min^−1^. Mobile phases were filtered through a 0.45 µm Nylon 66 membrane (Supelco, Oakville, ON, Canada) and degassed for 20 min in a VWR Scientific Aquasonic model 75HT (West Chester, PA, USA) ultrasonic bath before use.

Further details regarding the chromatographic methods employed in this study are provided in the Supplementary Information.

For analysis, 5.0 µL of final extracts were injected onto the column in partial loop mode. The injection system was cleaned by flushing and washing (1 mL each) the syringe with ACN/H_2_O (70/30, v/v) after each injection.

Chromatographic data acquisition, peak integration, and quantification were performed using Xcalibur software v.2.0.7 (Thermo Fisher Scientific, San Jose, USA).

Extractions via TF-SPME were performed with use of either a manual or an automated Concept 96 system (Professional Analytical System (PAS) Technology, Magdala, Germany).

### Design of SPME for negligible depletion extraction

The main objective of this study was to develop a high-throughput non-depletive SPME method that can be applied to small sample volumes for simultaneous determinations of free and total concentrations of target compounds of varying physicochemical properties.

To address this challenge, the experimental parameters required for measurements of free concentrations under undisturbed conditions had to first be considered. The criteria to obtain non-depletive conditions is given in equation 1, where V_s_ is the sample volume, V_f_ is the volume of the extraction phase, C_free_ is the free concentration of analyte in the presence of binding components, C_total_ is the total concentration of analytes, and K_fs_ is the distribution constant of the analyte between the extraction phase and the sample^10^.

1 $${V}_{s}{C}_{free}\,\rangle \rangle \,{K}_{fs}{V}_{f}{C}_{total}$$

For solid coatings, the volume of the extraction phase (V_f_) can be replaced with the total active surface area of the extraction phase (S_a_), and a similar criteria can be considered (see equation 2).

2 $${V}_{s}{C}_{free}\,\rangle \rangle \,{K}_{fs}{S}_{a}{C}_{total}$$

To achieve non-depletive conditions and enable several extractions from the same sample under the same conditions, the extracted amounts of analytes should be small enough so as to be negligible in comparison to the total amount of analyte present in a given sample. Usually, extraction of less than 5% of the unbound concentration is considered to result in negligible depletion. Since the sample volume is determined by the intended application, the most critical parameters to be tuned are the volume/active surface area of the extraction phase, and the extraction phase chemistry, which influences the distribution constant of a given analyte between the extraction phase and the sample, namely its K_fs_ value.

Since the selected volume of sample (100 µL) only enables a sampling depth of few millimetres in 96 well plates, the length of the SPME coating was limited to 1–2 mm. Furthermore, in view of the small sample volume in question, and considering that sequential extractions over a given time period are intended, prolonged extraction periods may result in substantial sample loss due to evaporation. Aiming to avoid sample evaporation, and hence changes in analyte concentration, consideration had to be given to extraction time, in that employed SPME extraction phases should facilitate short extraction times (less than 10 min)—thus necessitating rapid equilibration between the sampling device and solution. Consequently, another critical parameter investigated in terms of coating volume concerned the thickness of the employed coatings.

In SPME, the chemistry of the selected extraction phase crucially influences the types and amounts of analytes that can be extracted from a given matrix. In this regard, SPME offers a significant advantage over traditional methods for analysis of complex biological matrices, in that SPME can enable balanced coverage of a wide range of analytes. Essentially, compounds with low affinity for SPME extraction phases (e.g. polar compounds) typically also demonstrate low protein binding affinity, resulting in a high proportion of that chemical freely available in solution for extraction by the extractant. Therefore, the “lack of sensitivity” of the extractant is “balanced” by the high concentration that is available for extraction, namely the free concentration of a given analyte. In contrast, much lower fractions of compounds that are highly bound to proteins or other matrix components will be freely available in solution. However, as these compounds usually exhibit strong affinities for SPME phases, the low free concentration in solution is “balanced” by the high extraction efficiency of the extractant for these chemicals. In this way, SPME can provide balanced coverage of analytes of diverse properties.

As such, this study included optimization of coating chemistry for wide coverage of analytes, and the selection of a coating geometry suitable for non-depletive extraction conditions. Following optimization, validation of the final method was performed.

### Data Availability

All data generated or analysed during this study are included in this published article or in the Supplementary Information.

## Results and Discussion

### Short coated TF-SPME for negligible depletion extraction

#### Extractive phase selection

Polystyrene-divinyl benzene with a weak anion exchanger (PS-DVB-WAX), octadecyl silica (C18), and hydrophilic lipophilic balanced (HLB) particles, all of which have been previously demonstrated to provide good extraction performance in terms of range of analyte coverage^19,20^, were selected for evaluation in this study. In addition, pentafluorophenylpropyl (F5, PFP) particles were also included in the evaluation as a potential sorbent candidate for TF-SPME. Four different desorption solutions identified as good solvent mixtures in previous studies^20,25^ were also selected: ACN/MeOH/H_2_O (40/40/20, v/v/v), ACN/MeOH/H_2_O (40/40/20, v/v/v) with 0.1% FA, ACN/H_2_O (80/20, v/v) with 0.1% FA, and ACN/H_2_O (50/50, v/v). All mixtures were individually tested for all aforementioned extraction phases. Experimental details of this evaluation are provided in the Supplementary Information.

Polypyrrole-coated blades prepared based on the coating method described by Piri‐Moghadam *et al*.^26^ were also investigated as a possible relatively low analyte affinity extraction phase; however, this approach was not able to prevent depletion of analytes (results not shown).

Results shown in Supplementary Fig. S1 demonstrate that a combination of HLB-coated blades and a desorption solvent of ACN/H_2_O (80/20, v/v) with 0.1% FA provided good coverage and the lowest carry-over during the desorption step for the three model compounds, as compared to the other evaluated extraction phases.

#### Optimization of TF-SPME geometry

**Figure 1:**
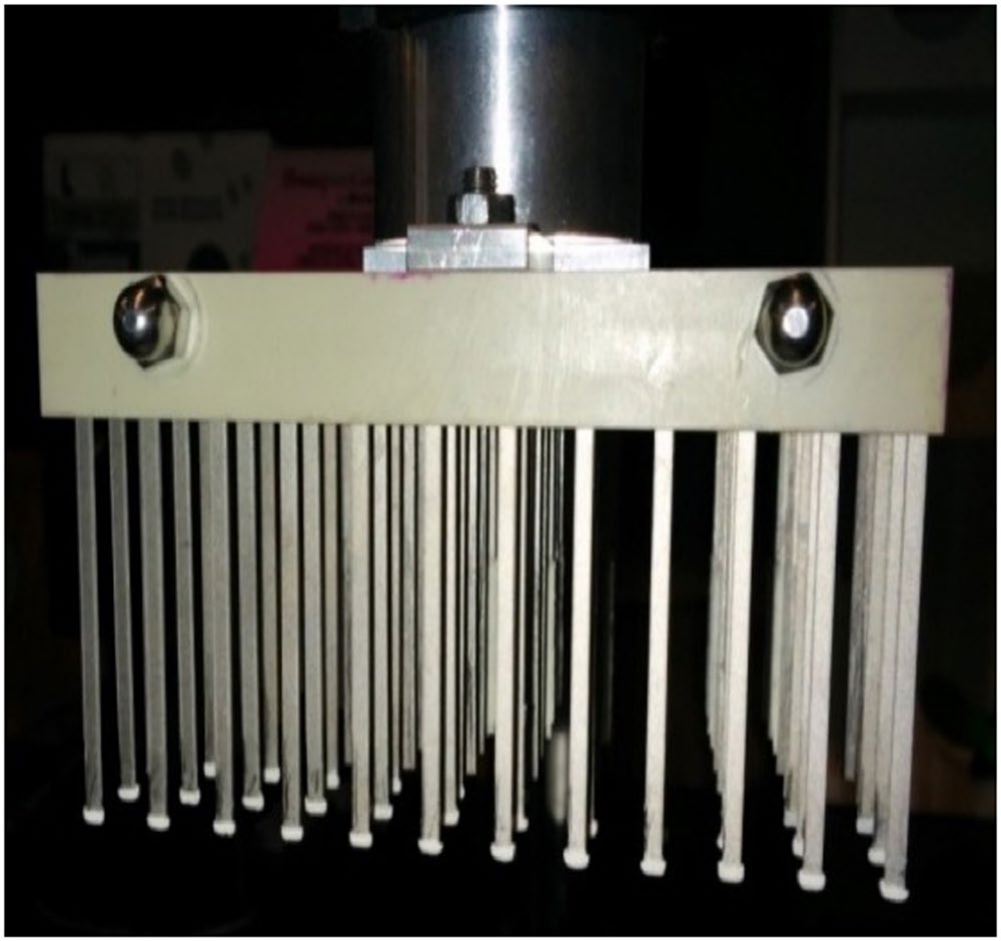
HLB TF-SPME blades with 1 mm coating length.

The length of the thin film coated on TF-SPME blades was adjusted from its typical length of 20 mm to 1 mm (Fig. 1) so as to ensure that the coating would be fully immersed in 100 µL of sample. For desorption, PCR plates with 200 µL volume capacities were used due to their narrow well design, which provided the lowest evaporation loss (*ca*. 10%) during a typical desorption step. General parameters for experiments using short-coated TF-SPME blades are provided in Supplementary Table S2.

#### Extraction time profile

The extraction time profiles of analytes extracted via HLB (60 µm particle diameter) coated TF-SPME are provided in Supplementary Fig. S2. As can be seen from the figures, under the tested experimental conditions, diclofenac and DEET reached maximum extraction levels after 60 min, while nicotine reached equilibrium after 30 min. However, the observed equilibria for diclofenac and DEET may be deceptive, as the attained recoveries for these compounds indicated exhaustive extraction. Given that the purpose of this study was to achieve negligible depletion, further investigations of extraction time points were not carried out for this configuration. The recovery results obtained at different extraction time points indicate that negligible depletion within the current geometry would only be possible under the shortest extraction time tested (1 min), which is far from equilibrium. Thus, this approach could potentially be applied for non-depletive extractions using short pre-equilibrium extraction times (≤1 min).

#### Figures of merits for TF-SPME method in binding-free matrix

Limits of quantification (LOQs) and regression coefficients of linearity were obtained from external SPME calibrations consisting of concentrations of 1.0, 5.0, 10.0, 25.0, 50.0, 100.0, 250.0, 500.0, 1000.0 and 5000.0 ng mL^−1^ for standards spiked in PBS. Internal standards (nicotine-d_4_, DEET-d_7_, and gemfibrozil or diclofenac-d_4_) were added to all samples in concentrations of 50 ng mL^−1^. Following SPME sampling, attained extracts were injected onto the LC-MS/MS. The LOQ for each analyte was chosen as the lowest level of standard that provided less than 20% deviation from their nominal concentration on the constructed SPME calibration curve. The calculated LOQ was 5.0 ng mL^−1^ for all compounds. Good correlations between the response of the extracted amounts and spike levels were observed. Intra-day, inter-day, and inter-blade precisions were evaluated for three levels of working concentrations (10.0, 100.0, and 1000.0 ng mL^−1^) in PBS. As can be seen in Table S3 (see Supplementary Information), all results showed good reproducibility, except for inter-day precision of nicotine and DEET extractions at the lowest tested concentration. However, it should be noted that the inter-day precision measurements were calculated based on the absolute recoveries of analytes on each day, which were determined against an external instrumental calibration curve. In this regard, precision could be further improved through the use of an external SPME calibration method.

#### Evaluation of the developed method for repetitive extractions in the presence of a binding matrix

The applicability of the method for repetitive extraction in the presence of a binding matrix was tested with the use of fetal calf serum (CS) as a sample matrix. For this purpose, calf serum was first spiked to contain 1.0 µg mL^−1^ of all analytes, then equilibrated on a shaker at 1200 rpm for 18 h at approx. 20 °C (room temperature). In total, 6 repetitive extractions, carried out every 30 or 45 min over a 4 h incubation period, were performed on the same samples. The selected combination of extraction sample volume, extraction phase, and coating geometry resulted in higher extraction values than expected, in particular for analytes characterized by moderate and high protein binding (PB) values (see Table 1 and Supplementary Fig. S3). This was especially evident for diclofenac, which has a reported plasma protein binding of more than 99%;^27^ recoveries after the first extraction indicated that the extraction was close to depleting the free available concentration. To illustrate this, for a sample spiked with 100 ng of analyte (as in this case), based on a PB of 99%, only 1 ng of the analyte would be free in solution, while the remainder molecules would be expected to be bound to plasma proteins; yet, for the currently discussed extractions, the extracted amount of diclofenac was 0.34 ng (i.e. resulting in an extraction of 34% of the free amount) for a single extraction under these conditions. However, repetitive extractions from the same sample showed recoveries similar to the first extraction. These observations suggest that the free concentration of the analyte would have been substantially depleted by the TF-SPME blades, causing the analyte protein binding equilibrium to shift in order to compensate for analyte depletion in the sample due to extraction. The outcome of these experiments demonstrated that the short-coated TF-SPME method is inadequate for achieving the ultimate goal of this study. Nonetheless, this method, based on a reduced geometry of TF-SPME, is well suited for high-throughput extractions of multi-residue samples from limited amounts of complex matrices in cases where only total concentration determinations in single-use samples are required.

**Table 1:** Extraction amounts from calf serum with developed TF-SPME method. Expected extraction amounts were calculated based on 5% depletion from the free concentration. Protein binding of DEET has not been reported (NR) in the literature. Extraction conditions: analyte concentration of 1000 ng mL^−1^, extraction time 30 sec, 30 min equilibration between extractions, desorption time 10 min.

	Nicotine	DEET	Diclofenac
Protein binding	<10%	NR	>99%
Concentration in sample ng mL^−1^	1000	1000	1000
Total amount (ng) in 100 µL	100	100	100
Expected free amount (ng) in 100 µL	90	—	<1
Extracted amounts (ng)	4.2	3.1	0.34
Expected extraction amount (ng)	4.5	—	0.05

Aiming to further reduce the coating volume of HLB coatings, thinner TF-SPME coatings were prepared via the previously mentioned spraying method, using 60 µm HLB particles. Although thinner coatings with various numbers of layers were prepared, depletion was still observed when coatings were applied towards extraction under the above described conditions. In view of this, a recently optimized dip coating methodology based on 5 µm HLB particles was used for preparation of ultra-thin coated blades (<10 µm). However, depletion and high recoveries were still observed for this coating, owing to the relatively large extraction phase present on the blades and the small sample volumes employed in this study.

#### Ultra-thin coated SPME fibers for negligible depletion extraction

**Figure 2:**
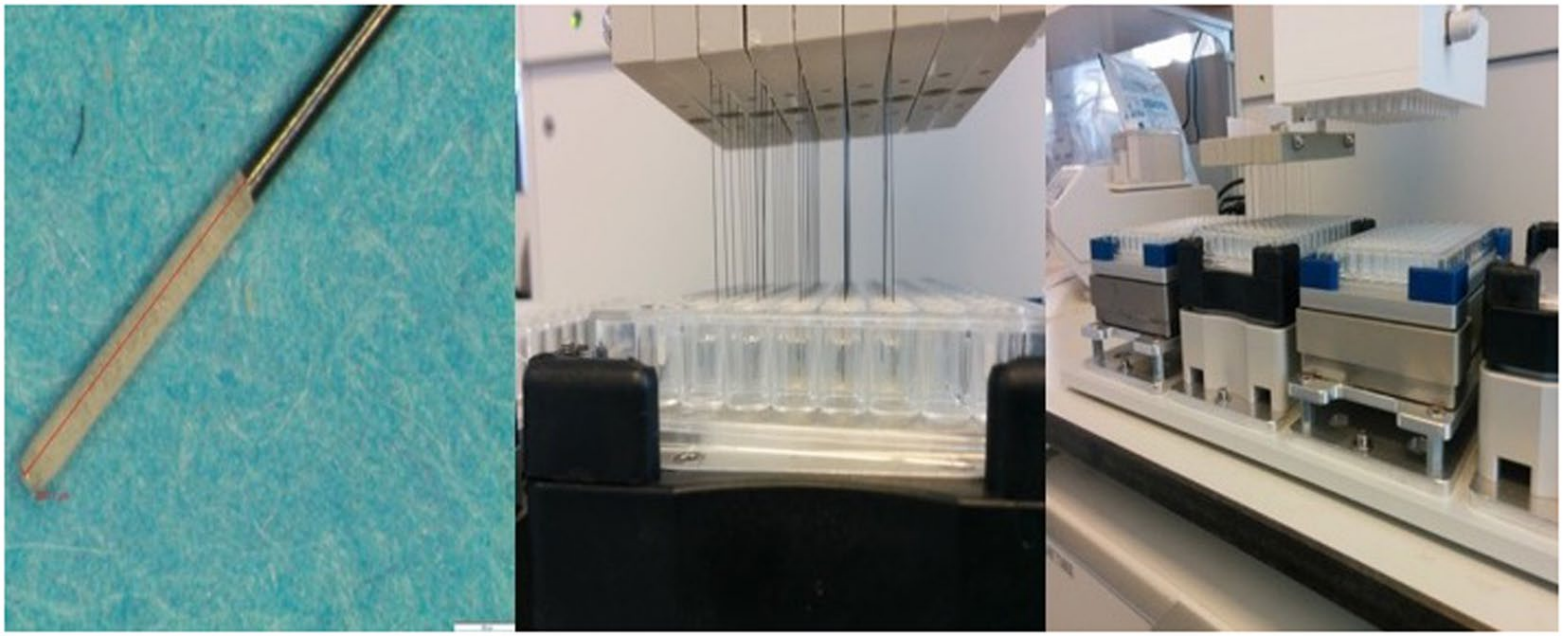
HLB coated nitinol fibers and automation of the fiber SPME for high throughput *in vitro* studies. Left figure illustrates an ultra-thin coated SPME fiber, middle figure shows a typical assembly of SPME fibers for high throughput extraction from biological sample, and right figure demonstrates automation of entire sample preparation protocol for *in vitro* non-depletive sampling.

As the blade geometry did not allow for sufficient reduction in the extraction phase volume, a new approach, using a sampling device consisting of a nitinol wire with a diameter of 200 µm, was devised. For this purpose, the thin coating method (Fig. 2) was applied, using 5 µm diameter HLB particles. This approach would theoretically ensure a wide analyte coverage by the selected HLB phase, while fast extraction and lower recoveries would be achieved due to the thin and miniaturized coating (2 mm coating length, <10 µm thickness) format, thus ensuring non-depletive extraction.

The manufactured ultra-thin coated (6 µm coating thickness) SPME fibers functionalized with HLB particles (5 µm in particle diameter) were evaluated via protein binding determinations, and repetitive extractions performed from the same sample. The general experimental steps were the same for each experiment, and are summarized below, while the parameters that may have varied between experiments are indicated in the related figure or table caption.

#### Protocol for fiber SPME

The SPME experimental workflow consisted of five major steps. First, prior to extraction, fibers were conditioned in 100 µL of desorption solution ACN/H_2_O (80/20, v/v), which was acidified to contain 0.1% (v/v) formic acid. The second step, occurring prior to extraction, involved submersing the fiber in 100 µL of ultrapure water to remove traces of solvent from the extraction phase. This step was performed for 10 seconds using 100 rpm agitation, with water temperature set to 37 °C in order to avoid a temperature gradient between the fiber and the sample, as this could influence the initial uptake of analytes. Following, extraction was performed on 100 µL of sample for 5 min, with samples thermostated to 37 °C, using 100 rpm agitation, prior to extraction. The fourth step consisted of a second rinsing of the fiber with 100 µL of water for 10 seconds to remove matrix components from the fiber. This step was critical, as it ensured that there were no traces of binding components remaining on the coating that could interfere with the quantitation of analytes extracted by the SPME fiber. To that end, stronger agitation (850 rpm) was applied in this step. Finally, the desorption step was conducted in 150 µL of desorption solution, which consisted of ACN/H2O (80/20, v/v) acidified to contain 0.1% (v/v) formic acid. For this step, a desorption time of 2 minutes at 400 rpm agitation was used. A second desorption step was sequentially performed to ensure that carry-over did not occur, as well as to serve as a pre-conditioning step prior to the next extraction. Fibers were subjected to three sequential dummy extractions from a blank sample matrix, using the aforementioned conditions, in order to stabilize their performance prior to their first use.

#### Determination of protein binding of selected analytes

The reliability of the new fiber-based SPME high-throughput method was tested by applying it to the determination of the protein binding (PB) percentages of the selected analytes. Two sets of samples covering a range of analyte concentrations were prepared; the first set of samples was prepared in phosphate buffered saline with a pH 7.4 (PBS), which did not contain binding components, while the second set was in a binding matrix (100% calf serum (CS)). Calibration graphs were constructed for both sets of samples by plotting the ratio of peak areas of analyte and internal standard (added at the desorption stage) against nominal analyte spike concentrations. The protein binding of each compound was calculated using the slope ratios of the linear portions of the calibration curves. The calculated PB values for DEET and diclofenac were 71% and 98%, respectively. The SPME calibrations were observed to significantly deviate from linearity for high concentration levels of analytes, even though extractions were performed under negligible depletion conditions (see Supplementary Fig. S4). This suggests possible saturation of the extraction phase for high concentrations. This phenomenon was more evident for nicotine, the most polar compound used in this study, as well as the analyte with the lowest affinity for the coating. In addition to the observed short dynamic ranges for analytes, the presence of analytes all together in the same sample at such high concentrations resulted in large standard deviations for extracted amounts that can be also related to the abovementioned possible saturation of the extraction phase for high concentrations. To decrease the possibility of saturation, and to increase the linear dynamic ranges, a second calibration experiment was performed with individually-spiked analytes. The results of this experiment showed that while the upper limits of the acceptable linear ranges had been doubled for all analytes, they were still limited to low µg mL^−1^ levels (data not shown). Chromatograms of the analytes obtained after extraction from calf serum (spiked to contain 100.0 ng mL^−1^ of analytes) using the developed method are shown in Fig. S5.

#### Repetitive extraction from 5% CS

Most *in vitro* assays are performed using human cell lines incubated in media consisting of buffers and additives, such as serum. Therefore, taking into consideration future potential applications, 5% CS in PBS was used in this study for repetitive extraction experiments to verify whether the method can be easily adapted for application in *in vitro* cell studies.

**Figure 3:**
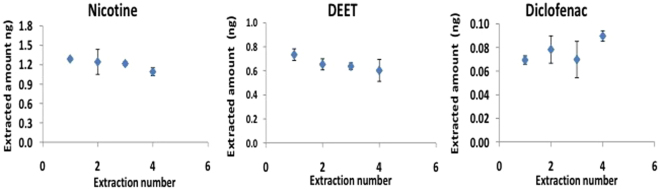
Repetitive extraction from 5% CS containing PBS. Extraction conditions: analyte concentration: 620 ng mL^−1^, extraction time 5 min, 30 min equilibration between extractions, desorption time 10 min.

As any evaporation from a small sample may result in substantial pre-concentration of matrix components, including of target analytes, the amount of water evaporation occurring from a 96-well plate during typical exposure periods was investigated in a model study. Aliquots of 100 µL of 5% CS in PBS were placed in 96 well plates and equilibrated at 37 °C for 20 min. Once the aliquots were equilibrated, the fiber holder was placed for 5 min in the sample, after which time a sample was removed from one of the wells and weighed. This was followed by another 30 min of equilibration and a second 5 min extraction, followed by the weighing of a sample from another well. This experiment was repeated 5 times. The plate was uncovered only during the 5-min extraction periods, but otherwise kept closed with a lid. At the end of the experiment, a significant decrease (*ca*. 30%) of the sample volume in the well was observed. However, no noticeable increases were observed for the amounts of analyte extracted over the duration of this experiment (see Fig. 3). Conversely, assuming evaporative pre-concentration did not occur (an ideal case scenario), then a declining trend in the extracted concentrations over repetitive extractions could be expected. Nevertheless, considering that neither of the possible trends were observed in terms of statistical significance between subsequent extraction points, the findings of this investigation point at a compensation between evaporation-based pre-concentration and gradual depletion with repetitive extraction. Therefore, it is important that both SPME external calibration and sampling be performed under the exact same conditions.

#### Comparison of the developed method to rapid equilibrium dialysis

The developed SPME method was further evaluated using a matrix consisted of 5% CS in PBS to mimic conditions typically used in *in vitro* cell assay applications. Protein binding percentages of the tested chemicals in 5% CS were determined using the developed high-throughput SPME fiber method, and compared to results obtained via rapid equilibrium dialysis (RED). SPME experiments were carried out as previously described, with the exception that the original binding matrix (calf serum) was replaced by 5% calf serum in PBS. Details regarding the comparative RED experiments, including used equipment, are provided in the Supplementary Information. For SPME experiments, PB values in 5% CS were determined as 98% for diclofenac, 38% for DEET, and 8% for nicotine; conversely, RED produced binding values of 91% for diclofenac, while DEET and nicotine values were determined as <10% binding. In general, these results indicated good agreement between the two methods.

#### Validation of the proposed high throughput fiber-SPME method

Since the final method is proposed for application in *in vitro* assays, PBS and 5% CS in PBS were selected as matrices for validation. External SPME calibrations were constructed by applying the developed method to both matrices, which were spiked with individual analytes to achieve concentrations of 1.0, 5.0, 10.0, 25.0, 50.0, 100.0, 250.0, 500.0, 1000.0, 2500.0, 5000.0, and 10000.0 ng mL^−1^. Each calibration point was extracted in triplicate, with subsequent instrumental analysis of SPME fiber extracts carried out by LC-MS/MS.

External SPME calibrations showed good linearity in both tested matrices. The limit of quantification (LOQ) was described as the lowest level of standard providing less than 20% deviation from the nominal concentration in back calculations from the constructed external SPME calibration curve. An LOQ of 5.0 ng mL^−1^ was calculated for nicotine and diclofenac in both matrices, while the LOQ of DEET was determined to be 10.0 ng mL^−1^ in both tested matrices. Furthermore, nicotine reached the upper limit of linearity at 2500.0 ng mL^−1^, while diclofenac and DEET showed adequate linearity up to the last testing point (10000.0 ng mL^−1^).

Method precision was evaluated based on inter-fiber and intra-fiber reproducibility, for which target analytes were spiked individually at various concentrations representing the low, mid, and high ranges of the calibration curves. Inter-fiber reproducibility was performed with 4 fibers (for each spike level) using PBS and 5% CS-containing PBS, which were both spiked separately with the test chemicals. Intra-fiber reproducibility was demonstrated by use of the same 3 fibers in 3 separate experiments employing the same conditions within a one-day period; thus, the attained results also demonstrated intra-day reproducibility. The spiking concentrations for diclofenac in PBS and 5% CS-containing PBS were 10.0, 100.0, and 1000.0 ng mL^−1^, and 50.0, 500.0, and 5000.0 ng mL^−1^, respectively. The spiking levels for nicotine in both matrices were 25.0, 100.0, and 1000.0 ng mL^−1^, while the spiking levels for DEET in PBS and 5% CS were 10.0, 100.0, and 1000.0 ng mL^−1^, and 25.0, 100.0, and 1000.0 ng mL^−1^, respectively. In general, the results summarized in Tables 2 and 3 reveal acceptable reproducibility for the test compounds at all tested levels, with a slightly higher RSD observed for diclofenac at 27% in PBS for the highest tested level (see Table 2). Considering that diclofenac has the highest log P value among the tested compounds, this compound would be expected to be more prone to secondary interactions with lab equipment, as well as present difficulties in its stabilization in matrices free of any stabilizer or binding components^28^. Calf serum provides an environment with possible stabilizing effects, which might explain why the obtained RSDs for diclofenac in this matrix were lower than those attained in PBS (see Table 3). On the other hand, since diclofenac has high protein binding affinity, even at serum levels of only 5%, the free concentration of the analyte should be relatively lower than its free concentration in the PBS solution. This resulted in a higher upper limit of quantification (ULOQ), which provided a better correlation between the constructed SPME calibration curves, and hence better reproducibility in the matrix that contained binding components.

**Table 2:** Precision of the method in PBS only matrix. For nicotine and DEET: Level 1: 25 ng mL^−1^, Level 2: 100 ng mL^−1^, Level 3: 1000 ng mL^−1^. For diclofenac: Level 1: 10 ng mL^−1^,Level 2: 100 ng mL^−1^, Level 3: 1000 ng mL^−1^. Inter-fiber reproducibility n = 4, intra-fiber reproducibility n = 3; experimental conditions: same as conditions given in the text, desorption volume: 0.1 mL.

Concentration (ng mL^−1^)	Intra-fiber reproducibility RSD (%)			Inter-fiber reproducibility RSD (%)		
	Nicotine	DEET	Diclofenac	Nicotine	DEET	Diclofenac
Level 1	2.7	0.4	21.7	7.0	3.6	7.2
Level 2	6.5	3.3	12.3	9.3	17.9	16.8
Level 3	12.4	9.6	27.4	1.2	7.2	2.3

**Table 3:** Precision of the method in 5% CS. For nicotine and DEET: Level 1: 25 ng mL^−1^, Level 2: 100 ng mL^−1^, Level 3: 1000 ng mL^−1^. For diclofenac: Level 1: 50 ng mL^−1^, Level 2:500 ng mL^−1^, Level 3: 5000 ng mL^−1^.Inter-fiber reproducibility n = 4, intra-fiber reproducibility n = 3; experimental conditions: same as conditions given in the text, desorption volume: 0.1 mL.

Concentration	Intra-fiber reproducibility RSD (%)			Inter-fiber reproducibility RSD (%)		
	Nicotine	DEET	Diclofenac	Nicotine	DEET	Diclofenac
Level 1	8.9	22.3	4.0	12.8	7.6	15.5
Level 2	10.1	5.1	1.9	13.7	4.6	9.9
Level 3	18.1	1.9	2.7	7.6	16.7	9.5

Method accuracy was assessed in 5% CS at the same analyte concentrations used for the precision evaluation. The relative recoveries from 5% CS were determined using SPME external calibration curves constructed in 5% CS; test samples were prepared in a batch of CS distinct from that used for preparation of the calibration curves. Extractions were performed in three replicates, and the average of these replicates at each calibration point was used to construct the calibration curve. Good agreement between extracted amount responses and spike levels, indicated in terms of percent relative error (RE%),was attained for all tested compounds (see Table 4), with the exception of nicotine extractions, which yielded a relative error of more than 20% at the mid-spike level.

**Table 4:** Accuracy of the method in terms of RE% in 5% CS. For nicotine and DEET: Level 1: 25 ng mL^−1^, Level 2: 100 ng mL^−1^, Level 3: 1000 ng mL^−1^. For diclofenac: Level 1: 50 ng mL^−1^, Level 2:500 ng mL^−1^, Level 3: 5000 ng mL^−1^. N = 3; experimental conditions: same as conditions given in the text, desorption volume: 0.1 mL.

Concentration	5% CS (RE%)		
	Nicotine	DEET	Diclofenac
Level 1	5.5	5.7	4.0
Level 2	−20.6	0.7	16.4
Level 3	−2.7	−12.9	10.7

Overall, the performance of the method indicated its suitability for analysis of both free and total concentrations of nicotine, DEET, and diclofenac in cell culture media. Although corrections with internal standards were not used for experimental errors at extraction stage, and only used to correct instrumental drifts and injection volumes, the obtained LOQs and RSD values were sufficient to show the robustness of the method for trace analysis. Further improvements to the attained LOQs could be achieved through the use of more suitable pre-concentration techniques; for instance, smaller desorption volumes or evaporation-reconstitution to smaller volumes could be incorporated to the proposed method. Alternatively, approaches stemming from recent advancements in the direct coupling of SPME to mass spectrometry^23,26^ could be considered to maximize system sensitivity.

## Conclusion

In this study, a high-throughput, non-depletive SPME-based method was developed for time-course determinations of free and total concentrations of a range of analytes in small sample volumes. The test chemicals (nicotine, DEET, and diclofenac) employed in this study were selected based on their diverse plasma protein binding characteristics.

Initially, a TF-SPME-based method using 1 mm HLB coating length was developed and tested. Despite the reductions in coating volume (length and thickness) that were achieved on a blade geometry, and the short non-equilibrium sampling times of 1 min, non-depletive sampling could not be achieved when applied to volumes typically employed in 96-well plate *in vitro* assays. However, the miniaturized TF-SPME method provides a means for high-throughput total concentration determinations from small samples, enabling a total sample preparation time of 11 min for 96 samples.

Aiming to carry out bioanalytical studies in micro-systems under non-depletive conditions, miniaturized, ultra-thin coated SPME fibers with a coating length of 2 mm and a coating thickness of 6 µm were developed. The fibers were successfully tested in a sample matrix consisting of 5% CS in PBS (a matrix commonly used in *in vitro* cell assays) to exemplify the potential use of the method in toxicokinetic and pharmacokinetic studies for repetitive non-depletive extractions from the same sample. The final method consisted of a 5 min extraction from 100 µL of sample at 37 °C, followed by a 10 s rinsing of matrix components with ultrapure water (100 µL) in room temperature; subsequently, desorption of extracted analytes was carried out in a desorption solution under 400 rpm agitation (110 µL) at room temperature for 2 min. The capability of the method to evaluate protein binding in 5% CS was successfully demonstrated and subsequently validated via rapid equilibrium dialysis. The final method meets the objectives of the study. Currently, the protocol is being further investigated for its applicability to simulated *in vitro* assay systems.

## Electronic supplementary material

Supplementary information
