# Supplementary material for: High-throughput analysis using non-depletive SPME: challenges and applications to the determination of free and total concentrations in small sample volumes
Source: Sci Rep. 2018 Jan 18;8:1167. doi: 10.1038/s41598-018-19313-1 (PMC5773572; doi:10.1038/s41598-018-19313-1)
Supplement: Supplementary file 1 — Supplementary information [file 41598_2018_19313_MOESM1_ESM.doc]

**Supplementary Information**

High-throughput analysis using non-depletive SPME: challenges and applications to the determination of free and total concentrations in small sample volumes

Ezel Boyacı^1^, Barbara Bojko^1^, Nathaly Reyes-Garcés^1^, Justen J. Poole^1^, Germán Augusto Gómez-Ríos^1^, Alexandre Teixeira^2^, Beate Nicol^2^ and Janusz Pawliszyn^1^*

^1^Department of Chemistry, University of Waterloo, 200 University Avenue West, Waterloo, Ontario, N2L 3G1, Canada

^2^Unilever U.K., Safety and Environmental Assurance Centre, Colworth Science Park, Sharnbrook Bedford MK441LQ, United Kingdom

**Table of Contents**

| Chromatographic methods……………………………………………………………….. | S-2 |
| --- | --- |
| Preparation of TF-SPME blades…………………………………………………………. | S-3 |
| Protocol for preliminary evaluations with 20 mm coated TF-SPME…………………… | S-4 |
| Rapid equilibrium dialysis……………………………………………………………….. | S-5 |
| Table S1. Optimized tuning parameters and SRM transitions of compounds in Thermo TSQ Vantage……………………………………………………………………………… | S-6 |
| Table S2. General experimental conditions applied with 1 mm coated HLB TF-SPME blades……………………………………………………………………………............... | S-7 |
| Table S3.Figures of merits of the method developed with short coated TF-SPME blades……………………………………………………………………………………… | S-8 |
| Figure S1. Selection of the coating and desorption solution for: a) nicotine; b) DEET; and c) diclofenac………………………………………………………………………….. | S-9 |
| Figure S2. Extraction time profiles of analytes with 1 mm HLB HLB-TF-SPME blades.. | S-10 |
| Figure S3. Repetitive extraction from CS with 1 mm HLB-coated TF-SPME…………... | S-11 |
| Figure S4. SPME calibration with HLB-coated fibers when all analytes are spiked together (a) nicotine, (b) DEET, and (c) diclofenac…………………………………......... | S-12 |
| Figure S5. Chromatograms of the analytes obtained after extraction from calf serum using the developed method………………………………………………………………. | S-13 |

**Chromatographic methods**

**Hilic method for nicotine.** An Ascentis Express HILIC column measuring 10 cm x2.1mm, 2.7 µm (Supelco) was used to separate nicotine and nicotine-d_4_. Chromatographic separation was achieved using solvent A, which was comprised of 10.0 mM ammonium acetate in water pH adjusted to 3.0 using formic acid, and solvent B, which consisted of ACN at a flow rate of 300 µL min^-1^. The gradient was initially set at 20% A and 80% B and was kept at this composition for 3 min; after 3 min had elapsed, it was changed to 80% A and 20% B in 0.1 min and held for 1 min. Next, the gradient composition was changed back to the initial condition (20% A and 80% B) in 0.9 min, and was kept constant for 2 min before the next injection.

**Reversed phase method for diclofenac and DEET.** For the separation of diclofenac, DEET, diclofenac-d_4_, and DEET-d_7_, an Ascentis Express F5 column measuring 5 cm x2.1mm, 2.7 µm (Supelco) was used. Chromatographic separation was achieved using solvent A, which was comprised of1.0 mM ammonium fluoride in water, and solvent B, which contained ACN at a flow rate of 300 µL min^-1^. The gradient was initially set to 80% A and 20% B and kept constant for 0.5 min; after 0.5 min had elapsed, the gradient was changed to 20% A and 80% B in 1 min and held constant for 1 min. Next, the gradient composition was changed back to the initial condition (80% A and 20% B) in 0.5 min, and held at this condition for 1 min before the next injection.

**Preparation of TF-SPME blades.** In order to ensure the coating’s stability, the blades were treated with concentrated HCl acid for 1 h. This step increases the surface roughness of the blades, which facilitates stronger particle-surface attachment. After treatment, the residual acid was washed off with a copious amount of water and the blade was dried in an oven for 30 min. PAN glue was prepared by gradually adding73 mL of DMF to 5.0 g of PAN in a 100 mL glass container. The complete dissolution of PAN in DMF was obtained after the mixture had been left to sit in a GC oven for 1 h at 90 °C. During the heating step, the lid of the jar was left slightly open to prevent any pressure build up. After 1h, the jar was placed in a fume hood to cool down to room temperature. Once the glue had cooled, 10 mL were mixed with 1.0 g of the extraction phase particles in a sprayer flask; to facilitate easy spraying, 3 mL of DMF was added to the final mixture. The flask-type sprayer was connected to an N_2_ line, and the flow was adjusted to produce a homogeneous spray of the material. Next, 20 mm or 1 mm of the bare blades were covered with a thin layer of the slurry and dried in a GC oven for 2 min at 150 °C. The same spraying and drying steps were repeated 10 times to get the final coating.

**Protocol for preliminary evaluations with 20 mm coated TF-SPME.** At the beginning of the study that evaluated the various extraction phases, 20 mm coated blades were used. For the extraction of analytes, a manual Concept 96 kit was selected. Extractions were performed at 850 rpm using 1.5 mL of PBS that had been spiked with analytes. After extraction, a quick (10 sec) rinsing step with 1.5 mL of ultrapure water was carried out to remove loosely-attached salt from the blades. Desorption was achieved in 1.5 mL of desorption solution at 850 rpm agitation.

Gemfibrozil, nicotine-d_4_, and DEET-d_7_ were used as internal standards by spiking the samples at a concentration of 50.0 ng mL^-1^; this was done in order to correct for variations in the sample-preparation step, as well as to compensate for instrumental and injection variations. Calibration standard solutions for LC-MS/MS were prepared in the desorption solution in a range of 0.1 to 100.0 ng mL^-1^, and were used to calculate the absolute amount of extracted analytes.

In order to shift the extraction amount of analytes to negligible depletion conditions, the coating length of the selected extraction phases was reduced from 20 mm to 1 mm. Any further modification in the extraction/desorption conditions are stated in the corresponding sections.

**Rapid equilibrium dialysis**

**Equipments.** The Rapid Equilibrium Dialysis (RED) Device Single-Use Plate with Inserts, 8K MWCO was obtained from Thermo Fisher Scientific (Rockford, IL, USA). Impact™ protein precipitation plates were purchased from Phenomenex (Torrance, CA, USA).

**Sample preparation for RED.** Stock standard solutions for each chemical were prepared at 1 mgmL^-1^ in DMSO and stored frozen (-20°C) until use. Each compound was prepared at 1000, 500, 100, and 10 ngmL^-1^ in treatment medium by serial dilution from stock standard solutions. Solutions of 500 and 50 ngmL^-1^ were also prepared in medium without FCS (time-to-equilibrium controls).

**RED protocol.** Each unit in the RED plate consists of two wells (a red/donor well and a white/acceptor well) separated by a permeable membrane with a molecular weight cut-off of 8K. Protein does not permeate membrane. Aliquots (400µL) of the spiked treatment medium solutions were added to the donor wells followed by the addition of600µL of blank medium to the acceptor cells.

A control was also performed by adding spiked media (500 and 50 ngmL^-1^) without protein to the donor well and blank media to the acceptor well. Equilibrium between the two wells is demonstrated if both wells have the same concentration at the end of the experiment. The experiment was performed in triplicate for all compounds and concentrations.

The RED plate was placed on an orbital incubated shaker at 800 rpm and 37 °C. After approximately 5h, 100 µL of sample was transferred into a protein precipitation plate along with 400 µL of internal standard solution (ACN). The plate was then agitated in the orbital shaker for 2 min and filtered under reduced pressure. Filtered samples were collected in an analysis plate and stored frozen until they were analyzed by LC-MS/MS.

**Table S1.** Optimized tuning parameters and SRM transitions of compounds in Thermo TSQ Vantage

| Compound name | Chemical  structure | Parent mass  m/z | Product mass  m/z | Collision energy  (V) | S Lens  (V) |
| --- | --- | --- | --- | --- | --- |
| DEET | 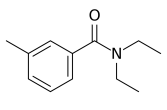 | 192.101 | 119.083 | 16 | 72 |
| DEET-d7 (IS) |  | 199.136 | 126.136 | 17 | 65 |
| Nicotine | [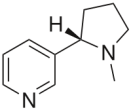](http://en.wikipedia.org/wiki/File:Nicotine.svg) | 163.000 | 130.107 | 20 | 59 |
| Nicotine-d4 (IS) | 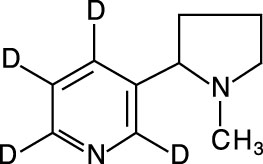 | 167.110 | 134.125 | 20 | 57 |
| Diclofenac | 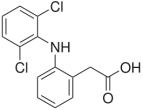 | 293.968 | 250.078 | 14 | 62 |
| Gemfibrozil (IS) | [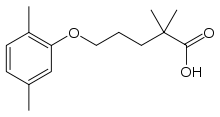](http://en.wikipedia.org/wiki/File:Gemfibrozil.svg) | 249.128 | 121.100 | 21 | 63 |
| Diclofenac-d4 (IS) |  |  |  |  |  |

**Table S2. General experimental conditions applied with 1 mm coated HLB TF-SPME blades**

| **Step** | **Conditions used** | **Reason of use** |
| --- | --- | --- |
| Pre-conditioning* | 80/20 (ACN/H2O) 150 µL, 5 min | Preparing the coating for extraction |
| Wash 1 | 10 s with 100 µL of pure water | Removes solvents remaining from 2^nd^ desorption, or pre-conditioning |
| Extraction | Temperature was adjusted to 37 °C, 100 rpm agitation, 100 µL of sample volume | - |
| Wash 2 | 10 s with agitation, 100 µL of pure water | Ensures the removal of loosely attached matrix components |
| Desorption | 80/20 (ACN/H2O) 110 µL for 10 min | Desorption of analytes |
| 2^nd^ desorption | 80/20 (ACN/H2O) 150 µL | Ensures elimination of carry-over |

***** This step was excluded for experiments where the blades were used directly after 2^nd^ desorption

**Table S3**.Figures of merits of the method developed with short coated TF-SPME blades

| Compound | LOQ  ng mL^-1^ | Intra-day precision  (n:3) | | | Inter-blade precision  (n:6) | | | Inter-day precision  (n:3) | | |
| --- | --- | --- | --- | --- | --- | --- | --- | --- | --- | --- |
|  |  | ng mL^-1^ | | | ng mL^-1^ | | | ng mL^-1^ | | |
|  |  | 10 | 100 | 1000 | 10 | 100 | 1000 | 10 | 100 | 1000 |
| Nicotine | 5.0 | 8.8 | 1.6 | 0.9 | 1.6 | 1.4 | 10.6 | 32.9 | 11.6 | 4.1 |
| DEET | 5.0 | 0.7 | 1 | 0.7 | 1.8 | 2.5 | 3.4 | 30.3 | 12.3 | 3.4 |
| Diclofenac | 5.0 | 0.8 | 0.9 | 2.2 | 2.9 | 2.3 | 6.0 | 11.7 | 6.7 | 11.4 |


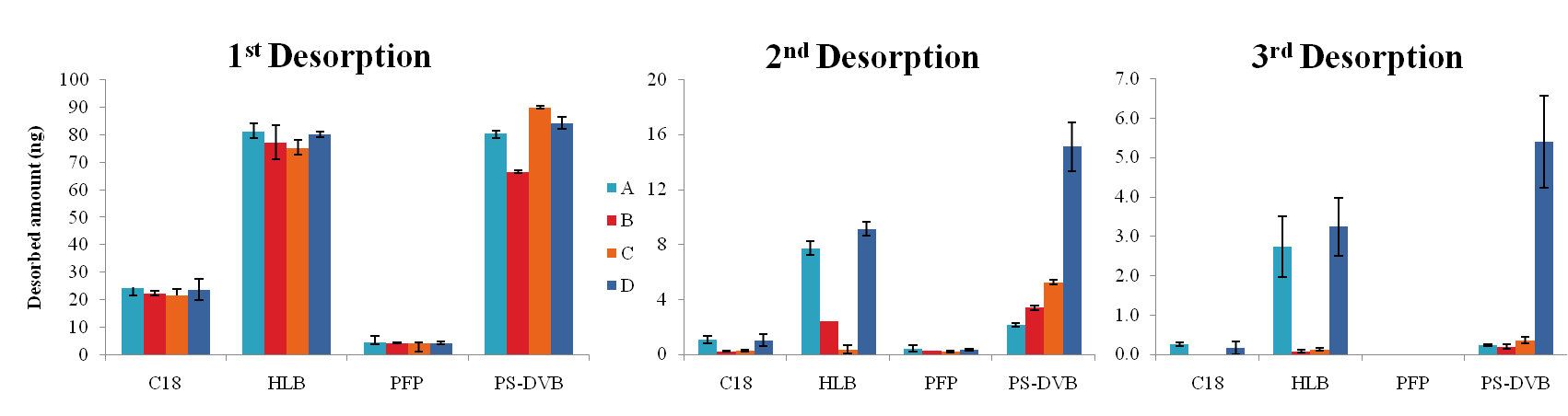


(a)


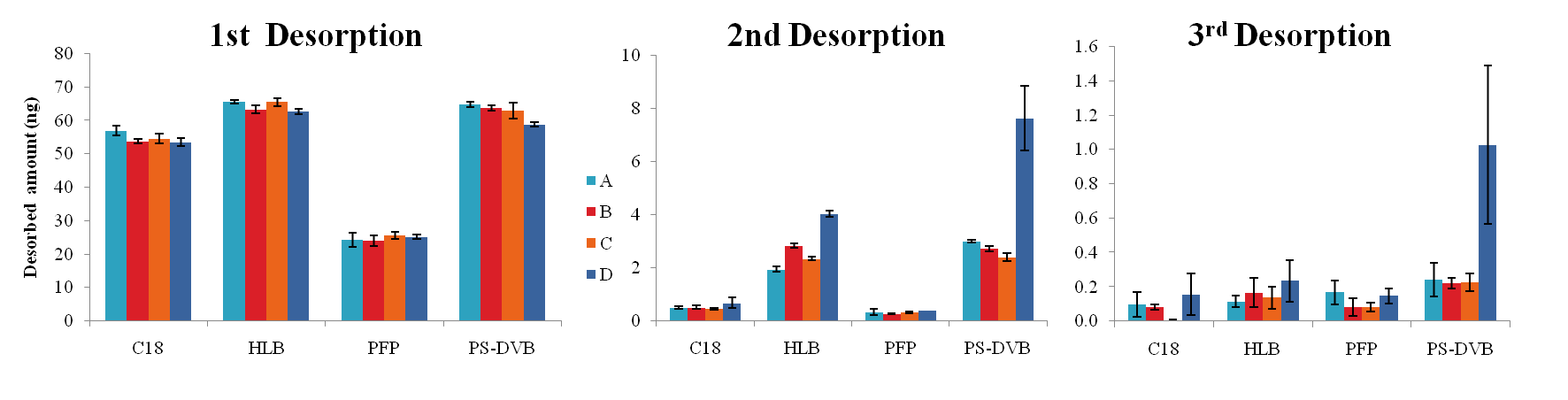


(b)


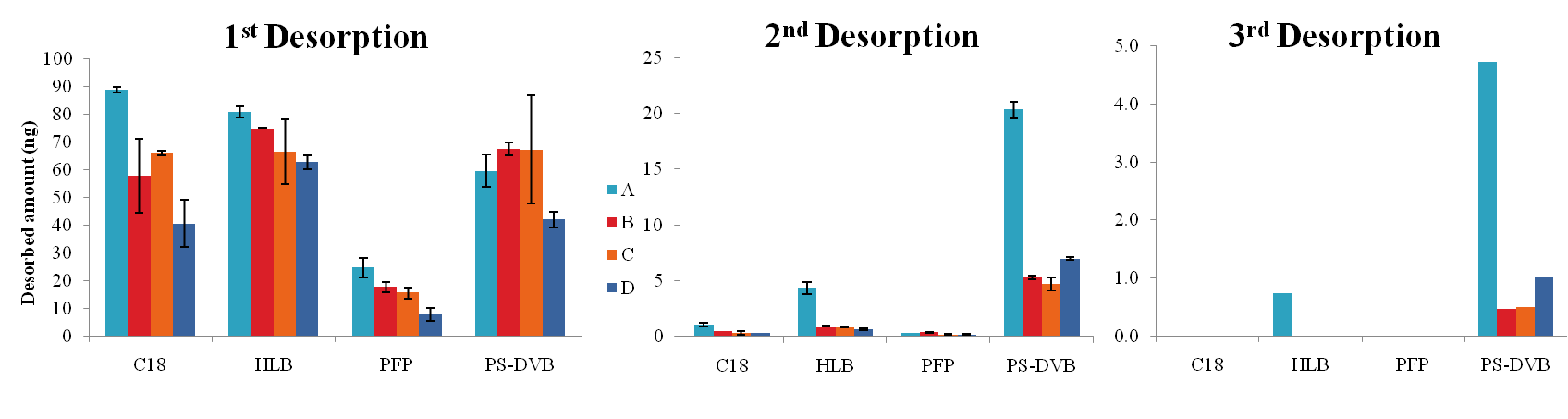


**Figure S1.** Selection of the coating and desorption solution for: a) nicotine; b) DEET; and c) diclofenac (Tested desorption solvents; A (light blue): ACN/MeOH/H_2_O (40/40/20, v/v/v); B (red): ACN/MeOH/H_2_O (40/40/20, v/v/v) with 0.1% FA; C (orange): ACN/H_2_O (80/20, v/v); and D (dark blue): ACN/ H_2_ O (50/50, v/v), Experimental conditions; eextraction and desorption times: 120 min; extraction and desorption volumes: 1.5 mL).


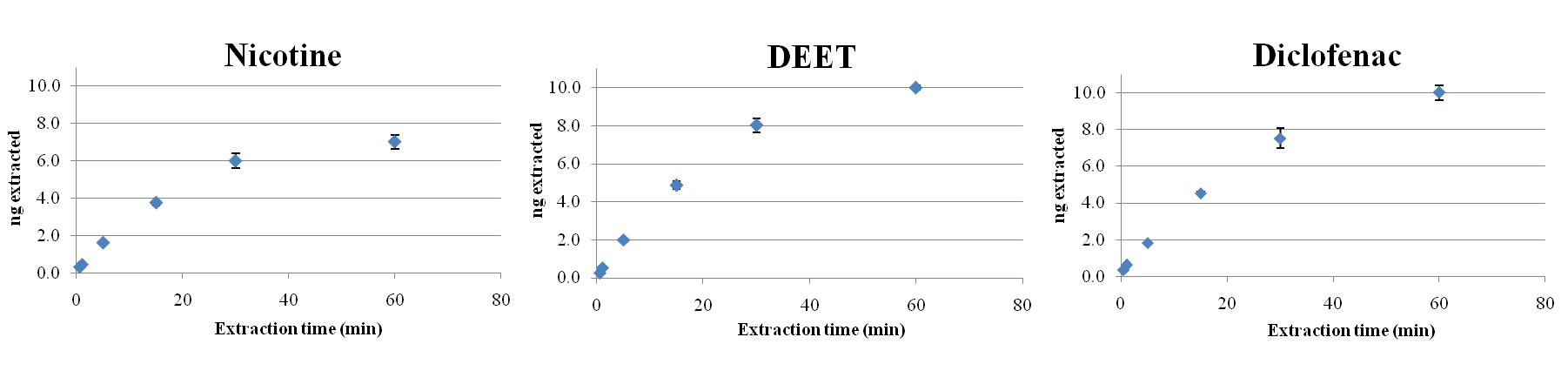


**Figure S2.** Extraction time profiles of analytes with 1 mm HLB HLB-TF-SPME blades (sample and its volume: 100 µL of PBS spiked to contain 100 ngmL^-1^of analytes; extraction temperature: 37 ºC; desorption solution and volume: 110 µL of ACN/H2O (80/20, v/v); desorption time: 10 min).

**Figure S3.** Repetitive extraction from CS with 1 mm HLB-coated TF-SPME (Extraction conditions: analyte concentration: 1000 ng mL^-1^; extraction time:30 sec; 30 min equilibration between extractions; desorption time 10 min)


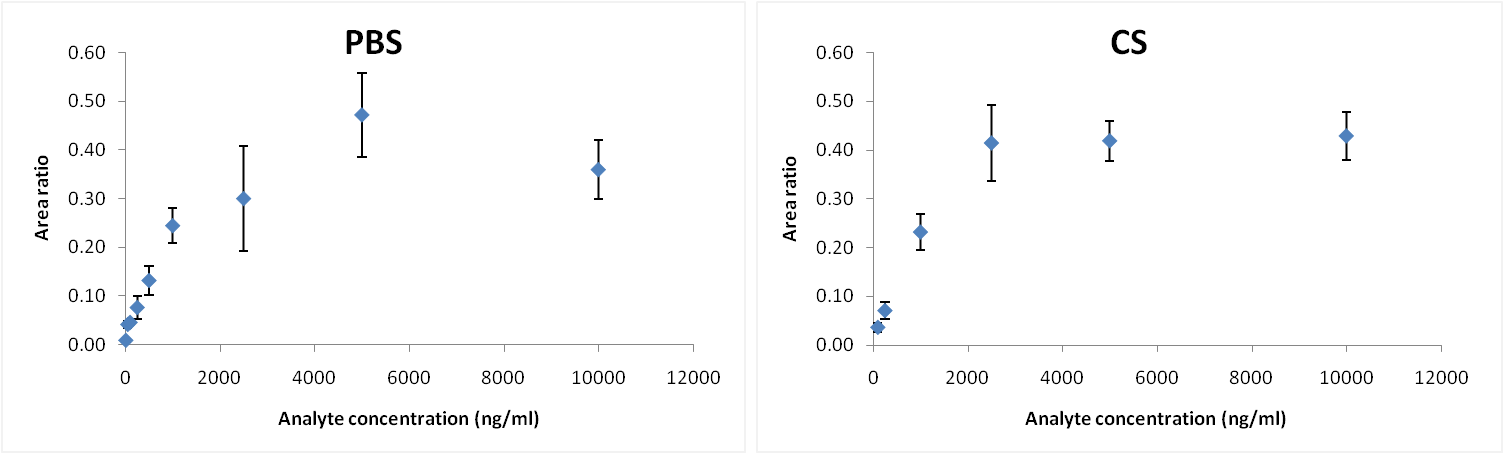


(a)


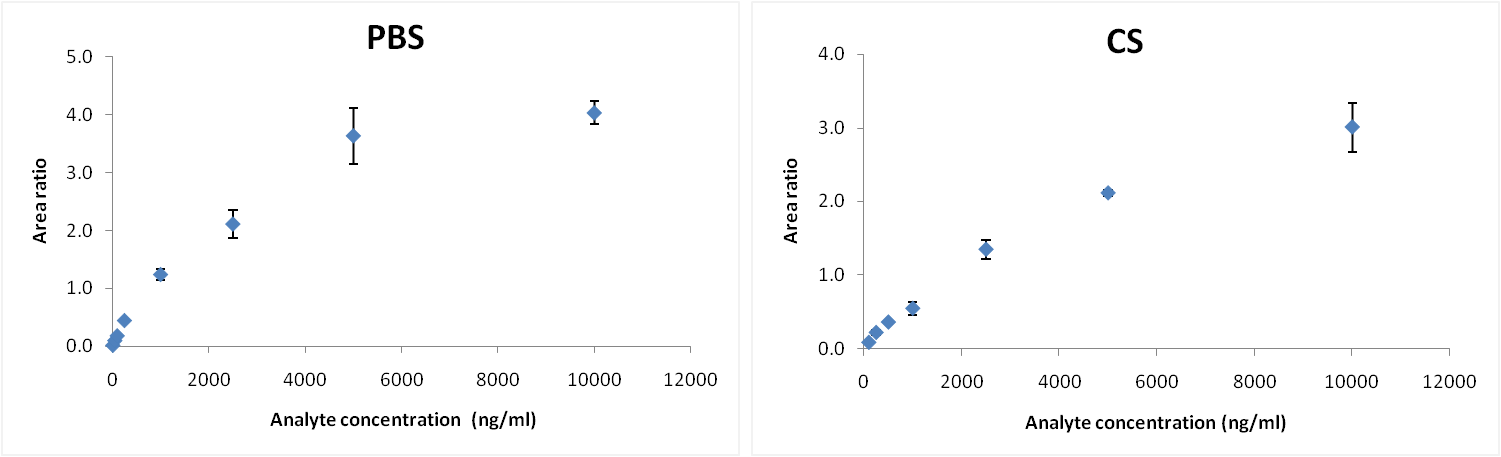


(b)


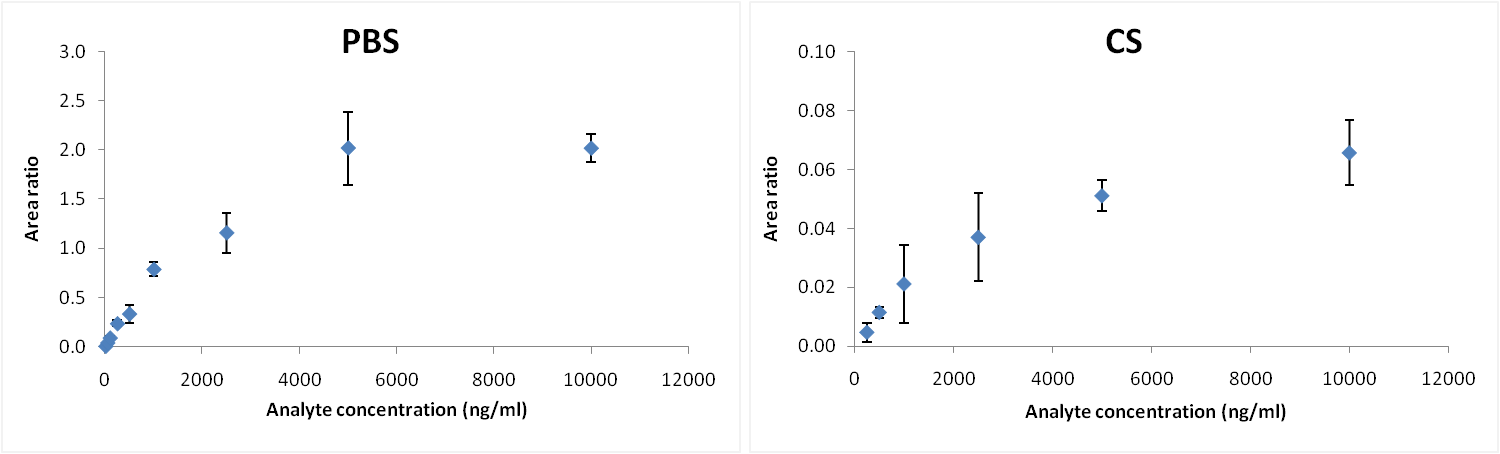


(c)

**Figure S4.** SPME calibration with HLB-coated fibers when all analytes are spiked together (a) nicotine, (b) DEET, and (c) diclofenac.

(a)

(b)

(c)

**Figure S5.** Chromatograms of the analytes obtained after extraction from calf serum using the developed method (a) nicotine, (b) DEET, and (c) diclofenac.
